# Global View on the Cytokinin Regulatory System in Potato

**DOI:** 10.3389/fpls.2020.613624

**Published:** 2020-12-21

**Authors:** Sergey N. Lomin, Yulia A. Myakushina, Oksana O. Kolachevskaya, Irina A. Getman, Ekaterina M. Savelieva, Dmitry V. Arkhipov, Svetlana V. Deigraf, Georgy A. Romanov

**Affiliations:** Timiryazev Institute of Plant Physiology, Russian Academy of Sciences, Moscow, Russia

**Keywords:** potato, cytokinin, two-component system, receptor, response regulator, gene expression, signaling chains, multistep phosphorelay

## Abstract

Cytokinins (CKs) were earlier shown to promote potato tuberization. Our study aimed to identify and characterize CK-related genes which constitute CK regulatory system in the core potato (*Solanum tuberosum*) genome. For that, CK-related genes were retrieved from the sequenced genome of the *S. tuberosum* doubled monoploid (DM) Phureja group, classified and compared with Arabidopsis orthologs. Analysis of selected gene expression was performed with a transcriptome database for the *S. tuberosum* heterozygous diploid line RH89-039-16. Genes responsible for CK signaling, biosynthesis, transport, and metabolism were categorized in an organ-specific fashion. According to this database, CK receptors StHK2/3 predominate in leaves and flowers, StHK4 in roots. Among phosphotransmitters, StHP1a expression largely predominates. Surprisingly, two pseudo-phosphotransmitters intended to suppress CK effects are hardly expressed in studied organs. Among B-type *RR* genes, *StRR1b*, *StRR11*, and *StRR18a* are actively expressed, with *StRR1b* expressing most uniformly in all organs and *StRR11* exhibiting the highest expression in roots. By cluster analysis four types of prevailing CK-signaling chains were identified in (1) leaves and flowers, StHK2/3→*StHP*1*a*→StRR1b/+; (2) shoot apical meristems, stolons, and mature tubers, StHK2/4→*StHP*1*a*→StRR1b/+; (3) stems and young tubers, StHK2/4→*StHP*1*a*→StRR1b/11/18a; and (4) roots and tuber sprouts, StHK4→*StHP*1*a*→StRR11/18a. CK synthesis genes *StIPT3*/*5* and *StCYP735A* are expressed mainly in roots followed by tuber sprouts, but rather weakly in stolons and tubers. By contrast, CK-activation genes *StLOGs* are active in stolons, and *StLOG3b* expression is even stolon-confined. Apparently, the main CK effects on tuber initiation are realized via activity of *StLOG1/3a/3b/7c/8a* genes in stolons. Current advances and future directions in potato research are discussed.

## Introduction

Potato tubers (*Solanum tuberosum* L.) are well known and widespread sources of food, feed, and technical substances (starches). Cytokinins (CKs), classical plant hormones, are known to promote potato tuber formation, at least in conventional *in vitro* systems (Palmer and Smith, 1970; Aksenova et al., 2000; Romanov et al., 2000; Cheng et al., 2020). CKs are also involved in the formation of artificial “tuberoids” on tobacco and tomato shoots (Guivarc’h et al., 2002; Eviatar-Ribak et al., 2013) as well as tuber-shaped rounded tumors on agrobacteria-infected plants (Dodueva et al., 2020). In addition to stimulating the formation of tubers, CKs contribute to their sprouting (Hartmann et al., 2011; Aksenova et al., 2013). The morphogenic effect of CKs certainly exploits their ability to induce cell divisions (Miller et al., 1955; Sakakibara, 2006; Romanov, 2009; Kieber and Schaller, 2014), although is not limited to this. All the above emphasizes the importance of CK regulatory system for such a valuable tuber-producing crop as potatoes.

Over the past decade, a prominent progress has been achieved in potato research. First of all, this concerns the sequencing of the complete genome of *S. tuberosum* group Phureja doubled monoploid DM1-3 516 R44 (DM) by the Potato Genome Sequencing Consortium [PGSC] (2011). Thereafter, a set of genes/proteins controlling tuberization was uncovered (Dutt et al., 2017; Hannapel et al., 2017). It became clear that the regulation of tuberization is based on a complex crosstalk between numerous hormonal and non-hormonal factors (Aksenova et al., 2012, 2014). In our research, we focused on the hormonal part of this regulatory network. On the basis of our experimental data (Kolachevskaya et al., 2015, 2017, 2018, 2019a) and data from recent literature, an updated hypothesis of hormonal regulation of potato tuberization was advanced (Kolachevskaya et al., 2017, 2019b), where CKs play an important role, especially at the tuber induction and initiation stages. Furthermore, the DNA sequence coding for CK receptors (sensor histidine kinase) and basic CK-signaling machinery were identified in the sequenced DM genome and analyzed by means of bioinformatics tools (Lomin et al., 2018b). In parallel, a suite of genes encoding sensor histidine kinases from tetraploid cultivar “Désirée” were cloned and expressed, giving rise to individual CK receptors. These receptors were studied in-depth, including their phylogenetics, conserved domains, 3D-structures, ligand-binding properties, organ-specificity of expression and responsiveness to CKs, regulatory *cis*-elements in their promoters, subcellular localization, homo- and heterodimerization, and mode of interaction with phosphotransmitters (Lomin et al., 2018b, 2020; Arkhipov et al., 2019).

Receptors are considered key proteins in hormone signaling, but they cannot signal by themselves. Regulation by any hormone *in planta* requires dozens of genes/proteins that function in various aspects of the given hormonal system. Like any other hormonal system, the CK regulatory system can be conventionally divided into a relatively conserved central part (CK signaling, synthesis, metabolism, and transport) and a more variable periphery—mainly CK-responsive regulatory genes (Bhargava et al., 2013; Brenner and Schmülling, 2015). For example, the central part of the model Arabidopsis plant distinguished by small genome size comprises, according to the latest estimates, some 80 genes (Supplementary Figure 1). Among them, 34 genes belong to the so-called two-component system (TCS) and constitute a signaling pathway termed multistep phosphorelay (MSP) from transmembrane receptors to primary response genes in the nucleus (Heyl and Schmülling, 2003; Mizuno, 2005; Kieber and Schaller, 2014, 2018; Pekárová et al., 2016, 2018; Arkhipov et al., 2019; Hallmark and Rashotte, 2019). Other involved genes have been classified as genes for CK synthesis (20 genes in total) (Kamada-Nobusada and Sakakibara, 2009), catabolism, and reversed inactivation (12 genes) (Schmülling et al., 2003; Hoyerová and Hošek, 2020), as well a transport (eight genes) (Liu et al., 2019). The CK regulatory system in potatoes is far less studied than in Arabidopsis. Here we intend to gain insight into the genome-wide composition and functioning of the central part of CK regulatory system in potatoes, with a particular focus on tuber formation. The prospects for using current knowledge to improve potato yield are discussed below.

## CK-Related Genes in Potatoes

Current molecular studies of potatoes are based on the sequenced DM genome representing the core genome of this widespread crop. Nevertheless, the size of even this minimal genome (844 Mb) is many times larger than the genome of the model Arabidopsis plant (135 Mb). However, this difference essentially disappears when we compare the numbers of protein-coding genes: 27,029 in *Arabidopsis thaliana* “Columbia” (Swarbreck et al., 2008) and 39,031 in DM potato (Potato Genome Sequencing Consortium [PGSC], 2011). In this case, the size of the one genome is now only reduced to only 1.44 times that of the other. Consequently, a large dissimilarity in numbers of genes of CK regulatory systems between these two species seems unlikely. Table 1 demonstrates that the core potato genome encodes orthologs of nearly all gene families involved in the central part of the CK system in Arabidopsis. And indeed, the sizes of orthologous gene families are rather close in Arabidopsis and potato (Supplementary Figure 1; Lomin et al., 2018b). For CK synthesis, potatoes possess 16 genes (compare to 20 in Arabidopsis), encoding six *isopentenyl adenine transferases* (*StIPT*), one cytochrome P450 monooxygenase *StCYP735A*, and nine *lonely guy* (*StLOG*) orthologs. For CK perception, potatoes have three receptor histidine kinases (StHK2–4) similarly to Arabidopsis; 8, 7, and 2 response regulators of class A, B, and C, respectively, as well as three cytokinin response factor (StCRF) proteins. In this regard, potatoes markedly differ from Arabidopsis in the proportion of TCS-like genes. In potatoes, half (three out of six) of the members of the phosphotransfer protein family (StHP) lack conserved phospho-accepting amino acids and are considered putative inhibitors of the MSP. By contrast, in Arabidopsis only one such member (AHP6, 1 from 6) is a true MSP inhibitor. In Arabidopsis, type B response regulators were divided into three groups, from which only ARR-Bs of the first group (ARR-BI) were proven to participate in CK signaling. Interestingly, ARR-Bs of these groups are not the closest neighbors on the phylogenetic tree but are interspersed with the APRR2 and APRR6 groups of pseudo-RRs. Similarly to Arabidopsis, most (7) of potato *RR-B* genes are homologous to RR-BI group, whereas three *StRR-B* genes are beyond this phylogenetic branch (Supplementary Figure 2A). The encoded three StRR-B proteins (StRR25, 27, and 28), along with conserved phospho-accepting aspartate, contain abnormal DD- and K-motifs in their receiver (REC-) domains (Supplementary Figure 2B), which most likely render these proteins inactive. Other potato non-canonical genes are listed in the Supplementary Table 1 as they hardly contribute to CK action. Notably, the potato genome contains genes encoding type C pseudo RRs (*PRR* type C) which are lacking in Arabidopsis. At least an essential portion of Arabidopsis PRR orthologs harbor CCT motif and take part in the photoperiodic flowering control unrelated to the CK system and to being subject to circadian rhythms (Mizuno, 2005). Any other role of the remaining PRRs (Supplementary Table 1) in CK action cannot be completely excluded but is very questionable.

**TABLE 1:** Proven or high-probable components of the CK regulatory system in DM potatoes.

Gene	GenBank			PGSC	
	Gene ID	Protein	Amino acids	Primary transcript	Location
**CHK**					
*StHK2*	LOC102591086	XP_015158747.1	1,263	PGSC0003DMT400015729	ST4.03ch07:44284533.44287254 R
*StHK3*	LOC102587294	XP_006352176.1	1,032	PGSC0003DMT400084727	ST4.03ch05:14358273.14361236 F
*StHK4*	LOC102603756	XP_006355050.1	992	PGSC0003DMT400075775	ST4.03ch04:2774233.2781847 R
**HK**					
*StCKI1*	LOC102594786	XP_006349947.1	765	PGSC0003DMT400011818	ST4.03ch12:60313883.60322789 F
*StETR1*	LOC102588584	XP_006349996.1/97.1	754/751	PGSC0003DMT400020285	ST4.03ch12:1112062.1121655 R
**HPt**					
*StHP1a*	LOC102590747	XP_006365269.1/70.1/71.1	151	PGSC0003DMT400081746	ST4.03ch01:60730670.60732462 R
*StHP1b*	LOC102603297	XP_006352793.1	152	PGSC0003DMT400051833	ST4.03ch06:59207849.59211570 F
*StHP4a*	LOC102589200	XP_006364721.1/XP_015159552.1	136/112	PGSC0003DMT400077451	ST4.03ch08:38158555.38161451 R
**PHPt**					
*StPHP4b*	LOC102584884	XP_015170906.1	137	PGSC0003DMT400047799	ST4.03ch11:42457958.42462239 R
*StPHP4c*	LOC102606269	XP_006343039.1/40.1	152		
*StPHP6*	LOC102601463	XP_006364219.1	156	PGSC0003DMT400001706	ST4.03ch03:56593561.56594955 R
**RR-B I**					
*StRR1a*	LOC102578736	XP_006363579.1/80.1	675	PGSC0003DMT400065835	ST4.03ch01:11998173.12002053 F
*StRR1b*	LOC102586468	XP_006345976.1	663	PGSC0003DMT400060506	ST4.03ch05:50113044.50119581 F
*StRR1c*	LOC102596771	XP_006349953.1	556	PGSC0003DMT400090747	ST4.03ch12:60090199.60093910 R
*StRR11*	LOC102593308	XP_015161764.1/67.1/68.8	481/581/581	PGSC0003DMT400031260	ST4.03ch05:11541271.11546411 F
*StRR14*	LOC102606335	XP_006355058.1/59.1	656/653	PGSC0003DMT400075907	ST4.03ch04:2709257.2714486 F
*StRR18a*	LOC102598455	XP_006343681.1	681	PGSC0003DMT400008290	ST4.03ch07:402301.406809 F
*StRR18b*	LOC102587717	XP_006350077.1	707	PGSC0003DMT400020233	ST4.03ch12:2023372.2028700 F
**RR-A**					
*StRR4*	LOC102602758	XP_015168830.1	248	PGSC0003DMT400058306	ST4.03ch05:6752023.6754796 R
*StRR9a*	LOC102590336	XP_006355595.1	163	PGSC0003DMT400007977	ST4.03ch02:30409061.30409639 R
*StRR9b*	LOC102588738	XP_015170232.1/33.1	214/211	PGSC0003DMT400007618	ST4.03ch04:24951499.24954972 f
*StRR9c*	LOC102599826	XP_006351272.1	226	PGSC0003DMT400076726	ST4.03ch10:58221853.58223696 F
*StRR9d*	LOC102601166	XP_006351276.1	226	PGSC0003DMT400076758	ST4.03ch10:58094625.58096547 R
*StRR15*	LOC102605280	XP_006344995.1	202	PGSC0003DMT400063187	ST4.03ch03:53710748.53712289 R
*StRR17a*	LOC102583233	XP_006357298.1	156	PGSC0003DMT400070964	ST4.03ch06:34954518.34956370 F
*StRR17b*	LOC102579353	XP_006358744.1	148	PGSC0003DMT400042922	ST4.03ch06:34247673.34249935 F
**RR-C**^1^					
*StRR22a*	LOC107059982	XP_015162643.1	137	PGSC0003DMT400092899	ST4.03ch03:23672034.23672546 R
*StRR22b*	LOC102580685	XP_006361623.2	115	PGSC0003DMT400096803	ST4.03ch03:37709010.37709447 R
**CRF**					
*StCRF1*	LOC102599019	XP_006343893.1	338	PGSC0003DMT400032058	ST4.03ch08:55617019.55618035 F
*StCRF3a*	LOC102605010	XP_006360182.1	374	PGSC0003DMT400006041	ST4.03ch06:38128680.38129804 F
*StCRF3b*	LOC102591238	XP_006350598.1	389	PGSC0003DMT400086922	ST4.03ch03:44865282.44866451 F
**ATP/ADP-IPT**					
*StIPT1a*	LOC102595379	XP_006360459	334	PGSC0003DMT400015411	ST4.03ch04:1541542.1542378 F
*StIPT1b*	LOC102604151	XP_006344131	345	PGSC0003DMT400037749	ST4.03ch05:2800989.2801840 R
*StIPT3*	LOC102579012	XP_006355868	323	PGSC0003DMT400002509	ST4.03ch09:46852324.46853283 R
*StIPT5*	LOC102599418	XP_006339408	330	PGSC0003DMT400083203	ST4.03ch01:61389842.61390834 R
**tRNA-IPT**					
*StIPT9*	LOC102605568	XP_006358948.1/49.1/XP_015169686.1	450/368/398	PGSC0003DMT400039795	ST4.03ch12:270799.276885 F
*StIPT2*	LOC102594223	XP_006349089/XP_015164944	467/466	PGSC0003DMT400068271	ST4.03ch11:39838731.39844810 R
**CYP735A**					
*StCYP735A*	LOC102603015	XP_006363117.1	517	PGSC0003DMT400032989	ST4.03ch02:41114766.41117388 F
**LOG**					
*StLOG1a*	LOC102584678	XP_006340180.1	228	PGSC0003DMT400020890	ST4.03ch11:41500955.41505580 F
*StLOG1b*	LOC102598045	XP_006365730.2	216		
*StLOG3a/LOG1*	LOC102581470	XP_006339070.1	220	PGSC0003DMT400027157	ST4.03ch10:54947968.54951688 F
*StLOG3b*	LOC102592821	XP_006354329.1	218	PGSC0003DMT400055525	ST4.03ch09:5646817.5650774 F
*StLOG7a/LOG2*	LOC102583076	XP_006348482.1	218	PGSC0003DMT400042349	ST4.03ch01:3283634.3289271 F
*StLOG7b*	LOC102587326	XP_006339321.1	217	PGSC0003DMT400072345	ST4.03ch10:56042745.56046560 R
*StLOG7c/LOG3*	LOC102592408	XP_006342033.1	225	PGSC0003DMT400009551	ST4.03ch04:70714467.70717363 F
*StLOG8a*	LOC102597227	XP_006351590.1	213	PGSC0003DMT400021223	ST4.03ch08:35250415.35253629 R
*StLOG8b*	LOC102595783	XP_015167145.1/XP_006354132.1	206/205	PGSC0003DMT400081828	ST4.03ch01:1308627.1312950 F
**CKX**					
*StCKX1a*	LOC102577758	NP_001275030.1	543	PGSC0003DMT400033123	ST4.03ch04:11108702.11111248 R
*StCKX1b*	LOC102605765	XP_006351290.1	536		
*StCKX3*	LOC102577888	NP_001275401.1	527	PGSC0003DMT400000752	ST4.03ch12:3650404.3656647 F
*StCKX5*	CKX4	NP_001274957.1	526	PGSC0003DMT400009621	ST4.03ch04:70221912.70227296 R
*StCKX6*	CKX3	NP_001275006.1/XP_015163315.1/16.1	533/509/533	PGSC0003DMT400017390	ST4.03ch01:64568740.64570801 F
*StCKX7a*	LOC102605861	XP_006367057.1	542	PGSC0003DMT400080680	ST4.03ch08:33930753.33933803 R
*StCKX7b*	CKX5	NP_001275121.1	513	PGSC0003DMT400080679	ST4.03ch08:33888758.33891903 R
**ABCG14**					
*StABCG14a*	LOC102579805	XP_006359961.1	646	PGSC0003DMT400004973	ST4.03ch08:47265148.47267889 R
*StABCG14b*	LOC102600750	XP_006361227.1/28.1	662/659	PGSC0003DMT400047547	ST4.03ch08:3599414.3602415 R
**ENT**					
*StENT3a*	LOC102583617	XP_006347184.1	415		
*StENT3b*	LOC102601592	XP_006347157.1/XP_015164176.1	421	PGSC0003DMT400057543	ST4.03ch02:35070810.35072418 R
*StENT3c*	LOC102592247	XP_006352597.1/98.1	448/421	PGSC0003DMT400029352	ST4.03ch10:520474.523484 F
*StENT3d*	LOC102604121	NP_001275018.1	418	PGSC0003DMT400059645	ST4.03ch02:10149698.10151875 F
**PUP**					
*StPUP1_1*	LOC102593174	XP_006338103.1	354	PGSC0003DMT400005470	ST4.03ch04:64474151.64477355 F
*StPUP1_2*	LOC102592621	XP_006358992.1	358		
*StPUP1_3*	LOC102592834	XP_006338102.1	352	PGSC0003DMT400005467	ST4.03ch04:64481580.64482969 F
*StPUP1_4*	LOC102580279	XP_006366415.1	355	PGSC0003DMT400071864	ST4.03ch06:36382676.36385224 R
*StPUP1_5*	LOC102581289	XP_006364706.1	363		
*StPUP1_6*	LOC102590236	XP_006357923.1	341		
*StPUP1_7*	LOC102590918	XP_015169158.1	351	PGSC0003DMT400036604	ST4.03ch06:36309239.36311744 R
*StPUP1_8*	LOC102591268	XP_006357925.1	343		
*StPUP1_9*	LOC102580805	XP_006360266.1	364	PGSC0003DMT400045772	ST4.03ch12:55367629.55371917 F
*StPUP1_10*	LOC102581812	XP_006360267.2	363		

*Gene families (left column) are marked bold. Slash separated and underlined end numbers in the Protein column corresponds to different splice forms. ^1^This family contains non-expressing genes in the displayed organ set (*StRR22a*) or in the overall organism (*StRR22b*).*

We included in Table 1 non-CK receptor histidine kinases (CKI1, ETR1) as they may play a role in CK signaling. When one such kinase (AtCKI1) was spontaneously overexpressed, the mutated phenotype mimicked the effect of massive CK treatment (Kakimoto, 1996). The receiver domain of another histidine kinase, ethylene receptor ETR1, also can interact with MSP signaling intermediates acting downstream from CK receptors (Zdarska et al., 2019). The background TCS activity of these proteins seems to be sufficient to rescue the basic phenotype of Arabidopsis triple mutants lacking all three CK receptors and no longer responding to CKs (Higuchi et al., 2004; Nishimura et al., 2004; Riefler et al., 2006; Romanov, 2009). All this indicates the possible role of CKI1 and ETR1 in MSP signaling. In total, the estimated number of genes directly involved in CK signaling (MSP) in DM potatoes is 31.

For CK degradation, potatoes possess seven CKX orthologs (Table 1). Genes for putative CK conjugation are included only in Supplementary Table 1 because potato *StUGT*s have no direct homology to Arabidopsis *UGT* genes responsible for CK *O*- and *N*-glucosylation (Supplementary Figure 3). Also, genes encoding CK transporters (Liu et al., 2019)—*StPUP* (10), *StENT* (4), and *StABCG14* (2) [possibly also *StABCI19-21* and *StAZG1,2* (Kim et al., 2020; Tessi et al., 2020), see Supplementary Table 1]—have active orthologs in Arabidopsis and are obviously of particular importance. The exceptions are gene-orthologs of *PUP14* transporters of Arabidopsis that are missing in potatoes (Supplementary Figure 4). Overall, the central part of the CK regulatory system in the core potato genome comprises 70 genes, the number close to that in Arabidopsis (Supplementary Figure 1). Among the potato genes, 28 (40%) are TCS homologs, whereas the remaining genes are not TCS-related.

## The Expression Pattern of CK-Related Genes

**FIGURE 1:**
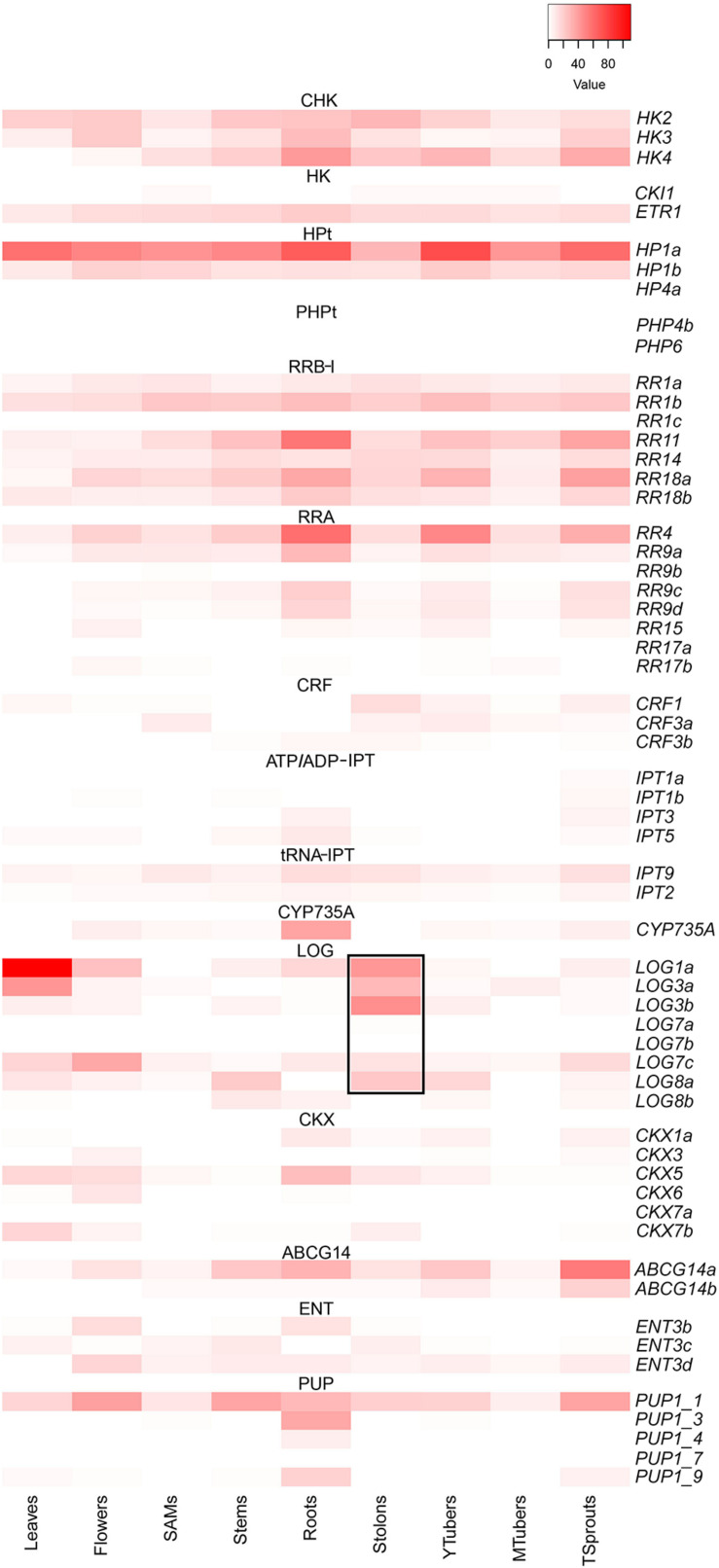
Expression pattern of cytokinin system genes of potato generated with the use of Heatmapper service (http://www.heatmapper.ca/expression/) (Babicki et al., 2016). The *LOG* gene expression pattern in the stolon is framed. SAMs, shoot apical meristems; YTubers and MTubers, young and mature tubers, respectively; TSprouts, tuber sprouts.

To elucidate the molecular events underlying potato growth, productivity and stress tolerance, the list of families of paralog genes is a useful but insufficient characteristic. Knowledge of absolute and relative spatiotemporal gene expression is necessary to address this issue. The expression pattern of genes involved in the CK action in the diploid potato line RH89-039-16^1^ is shown in Figure 1. Clearly, most of these genes are expressed differently depending on the organ and stage of development. Among CHK receptors, the expression of *StHK2/3* predominates in leaves and flowers, while *StHK4* is mostly expressed in roots. In the latter organ, *StHK2/4* genes are quite active. Among phosphotransmitters, *StHP1a* expression predominates in every organ, following by *StHP1b*, which is also expressed quasi-constitutively but to a much lesser extent. Interestingly, all three genes for the phosphotransfer-like proteins StPHP4b, 4c, 6, which are supposed to suppress CK signaling, are hardly expressed in RH89-039-16 line. As for RR-B transcription factors, *StRR1b* is expressed most uniformly in all organs, *StRR1a* acts similarly but much more weakly, and *StRR1c* is almost not expressed. Among all B-type *RR* genes, *StRR1a*, *StRR1b*, *StRR11*, and *StRR18a* are the most strongly expressed. Roots represent the site where almost all *RR-B* genes are expressed, dominated by *StRR11*.

By means of cluster analysis of the organ-specific expression of genes encoding receptors and StRRs type B (Supplementary Figure 5), we identified four types of prevailing signaling chains: (1) in leaves and flowers, StHK2/3→*StHP*1*a*→StRR1b/+; (2) in shoot apical meristems, stolons, and mature tubers, StHK2/4→*StHP*1*a*→StRR1b/+; (3) in stems and young tubers, StHK2/4→*StHP*1*a*→StRR1b/11/18a; and (4) in roots and tuber sprouts, StHK4→*StHP*1*a*→StRR11/18a.

Cytokinins synthesis genes *StIPT3/5* and *StCYP735A* are expressed mainly in roots (similarly to Arabidopsis). CK-perception and synthesis genes (*StHKs*, *StIPTs*, and *StCYP735A*) are also actively expressed in tuber sprouts, where *StHK4* transcripts prevail over transcripts of other CK receptor genes. A special group of CK-activation genes termed *StLOGs* are active in stolons, and *StLOG3b* expression is mainly restricted to this organ (Figure 1). It is noteworthy that organs (leaves, stolons) in which *StLOG* genes are strongly expressed, are mostly devoid of transcripts of other CK-synthesizing genes (*StIPT*, *StCYP735A*) (Figure 1). The above observations, based on the gene expression data^1^ for the diploid potato RH89-039-16, are generally consistent with organ-specific analysis of the transcriptome in the commercial tetraploid potatoes “Désirée” (Lomin et al., 2018b, and data not shown). However, the organ/tissue patterns of the expressed genes in different potato lines/cultivars (DM, RH89-039-16, “Désirée”) were not strictly identical, indicating some cultivar-specificity of gene functioning in potatoes. For example, RH89-039-16 and “Désirée” share similar organ-dependent expression patterns for *StHP1a, StPHP4b, StPHP6, StRR1b, StRR14, StRR18a, StRR9a, StCYP735A*, and *StABCG14a*, although marked differences in these patterns were observed for *StRR11, StRR9c, StIPT5*, and *StCKX3*. The expression patterns of remaining CK-related genes coincide moderately.

## Discussion

Here we present a global view on the CK regulatory system in potatoes. Generally, each hormonal regulatory system, in particular the cytokinin one (Romanov, 2009), includes complex units ensuring hormone biosynthesis, translocation, perception, and inactivation, as well as the primary response structures to targeted signal transduction. These units consist of corresponding proteins encoded by cognate genes. Here, the CK-related gene sequences were retrieved from the annotated core genome of the doubled monoploid DM1-3 516 R44 potato Phureja (the genes whose role in the CK system is proven or highly probable, Table 1) and thus represent the minimal gene set of the potato CK system. There is no doubt that commercial varieties of potatoes, mostly tetraploids, possess much many genes related to CK system. This is corroborated by data on the tetraploid “Désirée” variety, in which at least six authentic CK receptors have been identified (Lomin et al., 2018b), twice as many (3) CK receptors encoded by the DM genome. However, additional genes are close paralogs of the “core” genes, so the number of gene clades remains unchanged. Other genes that could theoretically acquire the status of CK “core” genes are listed in Supplementary Table 1, but their involvement requires detailed research and seems unlikely at this time.

In terms of nomenclature, we propose to follow the tradition of naming gene/protein according to the best homology to its Arabidopsis counterpart. Thereby, the potato genes were named according to their closest orthologs in Arabidopsis (see Supplementary Figures 2–4) in Supplementary Data. When many potato genes turned out to be orthologous to the same Arabidopsis gene, the former are marked with an additional letter at the end (a, b, c, etc.). Such nomenclature has already been used to designate potato CK-receptors (Lomin et al., 2012; Steklov et al., 2013) and other potato genes/proteins (Lomin et al., 2018b). The exceptions are *StPUP* genes since their homology to any of Arabidopsis *PUPs* is not obvious. In any case, this compiled gene set (Table 1) can serve as a convenient basis for studying genes, constituting the basic part of the CK system in potato.

As expected, most of CK-related enzymatic activities are encoded in the core potato genome by small (mostly 2–10 members) gene families. At the moment, 16 such families can be counted, of which eight families correspond to intracellular signal transduction (MSP), totally 31 genes encoding 41 proteins (Table 1). Among these proteins, only three (StHKs) have contact directly with hormonal ligands, while others obviously do not, though this has not been definitely proven. In fact, this part of the global CK system includes 28 authentic TCS homologs, which generate or affect signal transmission through His-Asp phosphorelay. The exceptions are proteins with degenerated TCS homology, which cannot directly participate in MSP because of structural deficiency. These non-functioning in MSP genes and proteins are evidently not *bona fide* members of the central part of the CK system. Some of them are displayed in Supplementary Table 1 containing not yet excluded but possible candidates for the CK system in potatoes. In fact, CKs almost monopolized the MSP system, using it as a signaling part of their global regulatory circuit. This statement is supported by the fact that every functional phosphotransmitter is promiscuous, i.e., is able to transmit “hot” phosphate from any CK receptor to any RR-B in the nucleus (Hutchison et al., 2006; Dortay et al., 2008; Lomin et al., 2018a; Arkhipov et al., 2019). Other hormones—in particular, ethylene, whose receptors are also TCS homologs—had to switch to a signal transduction pathway other than MSP (Pekárová et al., 2018).

Data on the relative expression of selected genes allowed us to outline the most plausible protein chains transmitting the CK signal from receptors to primary response genes. This corresponds to the activity of the prevailing potato MSP. We suggested four types of main signaling chains, which are delineated above. These chains are partially redundant, especially in relation to the transfer stage from the cytosol to the nucleus, where phosphotransmitter StHP1a predominates in all organs of the diploid potato. However, predominant receptors and B-type response regulators may differ depending on the organ, and this CK-signaling specificity should be taken into account when researchers manipulate the potato genome. To date, we consider the stolon as the most promising organ for engineering potato productivity and early maturation. Apparently, this is where the most important events leading to tuber initiation should take place. The most noticeable feature of the transcriptome of the CK system in stolons is the increased activity of the *LOG*-genes (*StLOG1/3a/3b/7c/8a*) without visible changes in the expression of other CK-related gene families. Moreover, the expression of *StLOG3b* is stolon-specific. In contrast, the activity of other CK synthesis genes, *IPTs* and *CYP735A*, was much weaker here than in other organs. Thus, stolons are likely sites where CK precursors (phosphoribosides) are exported and activated to functional CK bases by LOG enzymes. Recently, it was found that three *LOG* genes (*StLOG3a, 7a*, and *7c*) are activated by the tuberigen StBEL5, most likely through the direct interaction of this transcription factor with the corresponding *cis*-elements in the promoters of these genes (Sharma et al., 2016). It is noteworthy that the ectopic expression of only one of the tomato *LOG* genes was sufficient for the formation of tuber-like structures from the lateral meristems in tomato (Eviatar-Ribak et al., 2013). Thus, we assume that CKs are involved in the tuberization process through the activation of *LOG* genes by the StBEL5 pathway. The contribution of the stolon identity pathway is also possible due to the only partial overlap of *LOG* expression patterns induced by StBEL5 and inherent in stolons.

Collectively, since CKs were known to be implicated in many aspects of potato growth and productivity, the presented genome-wide data characterizing the CK-regulatory system of potatoes can be useful for the exploration and breeding of this important crop.

## Data Availability Statement

Publicly available datasets were analyzed in this study. This data can be found here: http://solanaceae.plantbiology.msu.edu/pgsc_download.shtml.

## Author Contributions

SL, YM, OK, IG, ES, SD, and DA presented and analyzed their experimental and/or bioinformatic results. SL prepared main illustrative materials. GR wrote the version of the manuscript. All authors contributed to the manuscript editing, developed the idea and outline of the manuscript, and read and approved the manuscript final version before submission.

## Conflict of Interest

The authors declare that the research was conducted in the absence of any commercial or financial relationships that could be construed as a potential conflict of interest.

## Footnotes

1 http://solanaceae.plantbiology.msu.edu/pgsc_download.shtml
