# Supplementary material for: Global View on the Cytokinin Regulatory System in Potato
Source: Front Plant Sci. 2020 Dec 21;11:613624. doi: 10.3389/fpls.2020.613624 (PMC7779595; doi:10.3389/fpls.2020.613624)
Supplement: Supplementary file 1 [file Data_Sheet_1.pdf]

SUPPLEMENTARY DATA for the article
"GLOBAL VIEW ON THE CYTOKININ REGULATORY SYSTEM IN POTATO"

Table S1. Low-probable components of the CK regulatory system in DM potato.
For special designations, see the caption to Table 1.

Gene	GenBank			PGSC	
	Gene ID	Protein	Amino acids	Primary transcript	Location
**HK**					
StHK1	LOC102596157	XP_006340754.1/55.1	1212/1211	PGSC0003DMT400009219	ST4.03ch02:39195290..39200917 R
StHK5	LOC102602523	XP_006355180.1	1015	PGSC0003DMT400075949	ST4.03ch08:44698892..44706453 F
**StRR**					
StRR25	LOC102598225	XP_015158676.1/80.1/83.1	353/336/331		
StRR26	LOC107059047	XP_015160928.1	202		
StRR27	LOC107060895	XP_015164912.1	371	PGSC0003DMT400085481	ST4.03ch11:38951813..38954419 R
StRR28	LOC107061394	XP_015166054.1	408		
StPRR29	LOC107061393	XP_015166053.1	362		
StPRR					
StPRR10a	LOC107061966	XP_015167530.1	260		
StPRR10b	LOC107063451	XP_015170840.1	217	***PGSC0003DMT400035788***	ST4.03ch07:12635684..12637807 F
StPRR10c	LOC107063452	XP_015170841.1	568		
**PRR6**					
StPRR6a	*LOC107063474*	*XP_015170872.1*	*611*	***PGSC0003DMT400086317***	*ST4.03ch07:6824808..6828625 F*
StPRR6b	LOC107062591	XP_015168890.1	465	PGSC0003DMT400012426	ST4.03ch06:48439578..48443405 R
StPRR6c	***LOC107061239***	***XP_015165596.1***	***653***	***PGSC0003DMT400090314***	***ST4.03ch03:45418057..45426000 F***
**PRR2**					
StPRR2a	LOC102599201	XP_006358579.1	560	PGSC0003DMT400062232	ST4.03ch08:50039181..50047582 R
StPRR2b	LOC102603798	XP_006361171.1/72.1/XP_015170639.1	554/554/528	PGSC0003DMT400068374	ST4.03ch06:44521328..44525386 F
**(P)RR-C**					
StPRR22a	***LOC107058083***	***XP_015158885.1***	***186***	***PGSC0003DMT400089551***	***ST4.03ch11:43091660..43092286 R***
StPRR22b	LOC107058085	XP_015158886.1	184	***PGSC0003DMT400086031***	ST4.03ch11:43117527..43118152 R
**(P)RR-CCT**					
StPRR1a	LOC102593655	XP_006364578.1	552	PGSC0003DMT400050252	ST4.03ch03:55735654..55741110 R
StPRR1b	LOC102582324	XP_006354770.1	549	PGSC0003DMT400083086	ST4.03ch06:51539409..51545194 R
StPRR3	LOC102583251	XP_006340553.1	783		
StPRR7	LOC102590489	XP_006363680.1/XP_015159036.1	729/585		
StPRR5a	LOC102589008	XP_006347516.1	680	PGSC0003DMT400001574	ST4.03ch03:46394283..46398400 F
StPRR5b	LOC102603187	XP_006352628.1	640	PGSC0003DMT400029402	ST4.03ch10:124572..130712 F
**CRF**					
StCRF7	LOC102598554	XP_006339673.1	300	PGSC0003DMT400000274	ST4.03ch01:73088486..73089388 F
StCRF8	LOC102578223	XP_006350890.1	248	PGSC0003DMT400078294	ST4.03ch06:57680479..57681225 R
**UGT76C**					
StUGT76			426 (PGSC)	PGSC0003DMT400055856	ST4.03ch03:33263486..33267729 R
**UGT85A1**					
StUGT85_1	LOC102582137	XP_006360268.1	486		
StUGT85_2	LOC102582803	XP_015170249.1	457	PGSC0003DMT400022063	ST4.03ch12:55326324..55327888 R
StUGT85_3	LOC102606387	XP_015158860.1	491	PGSC0003DMT400079198	ST4.03ch04:63845363..63846928 R
StUGT85_4	LOC102578390	XP_006363269.1	485	PGSC0003DMT400079201	ST4.03ch04:63872945..63874489 R
StUGT85_5	LOC102578731	XP_006363270.1	482	PGSC0003DMT400079203	ST4.03ch04:63886497..63888033 R
**ENT**					
StENT1	LOC102596988	XP_006348522.1	415	PGSC0003DMT400042188	ST4.03ch01:3695135..3698857 R
StENT8	LOC102605898	XP_006358278.1	419	PGSC0003DMT400079394	ST4.03ch07:4548953..4550865 F

**PUP**					
StPUP5	LOC102585453	XP_006343643.1	369	PGSC0003DMT400028938	ST4.03ch07:888050..889120 F
StPUP4_1	LOC102596411	XP_006364822.1	424	PGSC0003DMT400035529	ST4.03ch02:37929581..37930855 R
StPUP4_2	LOC102581882	XP_006352647.1	381		
StPUP4_3	LOC102582539	XP_006352648.2	352		
StPUP11_1	LOC102584861	XP_006354634.1	364	PGSC0003DMT400004320	ST4.03ch01:68399343..68400452 R
StPUP11_2	LOC102589062	XP_006358633.1	376	PGSC0003DMT400004651	ST4.03ch08:50218492..50220455 R
StPUP11_3	LOC102579572	XP_015169503.1	379	PGSC0003DMT400004654	ST4.03ch08:50209702..50210610 R
StPUPA	LOC102595253	XP_015167931.1/XP_006355609.2/610.1	377/375/377	PGSC0003DMT400025122	ST4.03ch02:30257580..30259715 R
StPUPB	LOC102595916	XP_006355612.1	392	PGSC0003DMT400025123	ST4.03ch02:30216674..30217941 R
StPUPC	LOC102594255	XP_006355608.1	386	PGSC0003DMT400025116	ST4.03ch02:30294422..30297715 R
StPUPD	LOC102579559	XP_006356083.1	379	PGSC0003DMT400035010	ST4.03ch03:789222..793736 R
**AZG**					
StAZG1	LOC102605023	XP_006361316.1/XP_015170700.1	575	PGSC0003DMT400074766	ST4.03ch06:31801108..31802025 F
StAZG2	LOC102581878	XP_006351463.1	539	PGSC0003DMT400039400	ST4.03ch03:51104049..51105668 F
**ABCI**					
StABCI19/21	LOC102594788	XP_006351662.1	286	PGSC0003DMT400026695	ST4.03ch09:49992798..49996316 R
StABCI20	LOC102589975	XP_006344794.1	329	PGSC0003DMT400007215	ST4.03ch01:78454127..78456996 F

Non-expressing genes in the displayed organ set or in the overall organism are marked in red italics or black bold italics, respectively. Recently revealed ABCI and especially AZG-type CK-transporters are real candidates for transferring in Table 1.

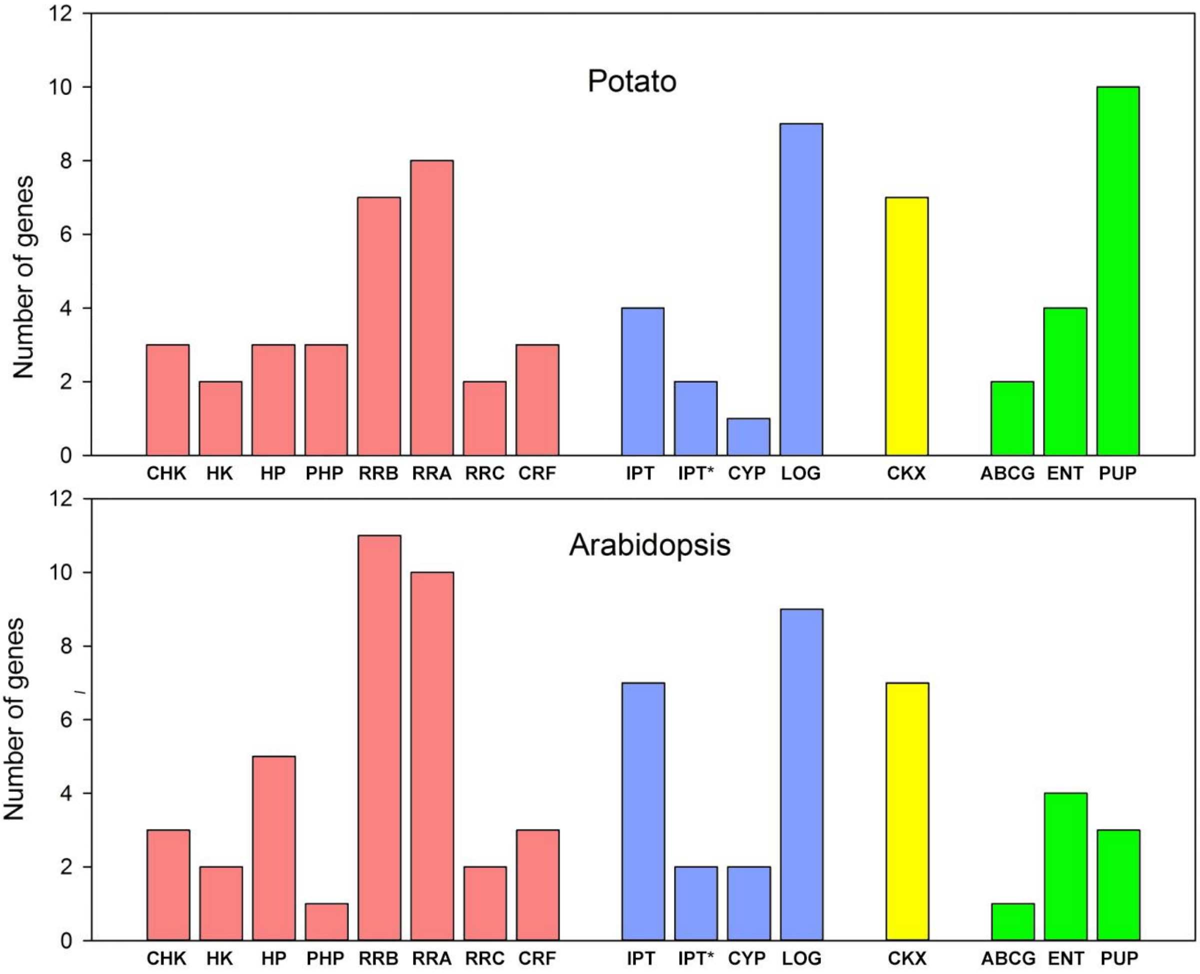

Fig. S1. Profiles of gene families related to the central part of CK regulatory systems of potato and Arabidopsis.

The genes families are (from left to right): CHK receptors; Histidine kinases; His-containing phosphotransmitters; His-containing pseudo-phosphotransmitters; Type B response regulators; Type A response regulators; Type C response regulators; CK response factors; Gap; IP-transferases (ATP/ADP); IP-transferases (tRNAs); CYP735A monooxygenase; LOG phosphoribohydrolases; Gap; Cytokinin oxidases/dehydrogenases; Gap; ABCG14 transporters; ENT transporters; PUP transporters.

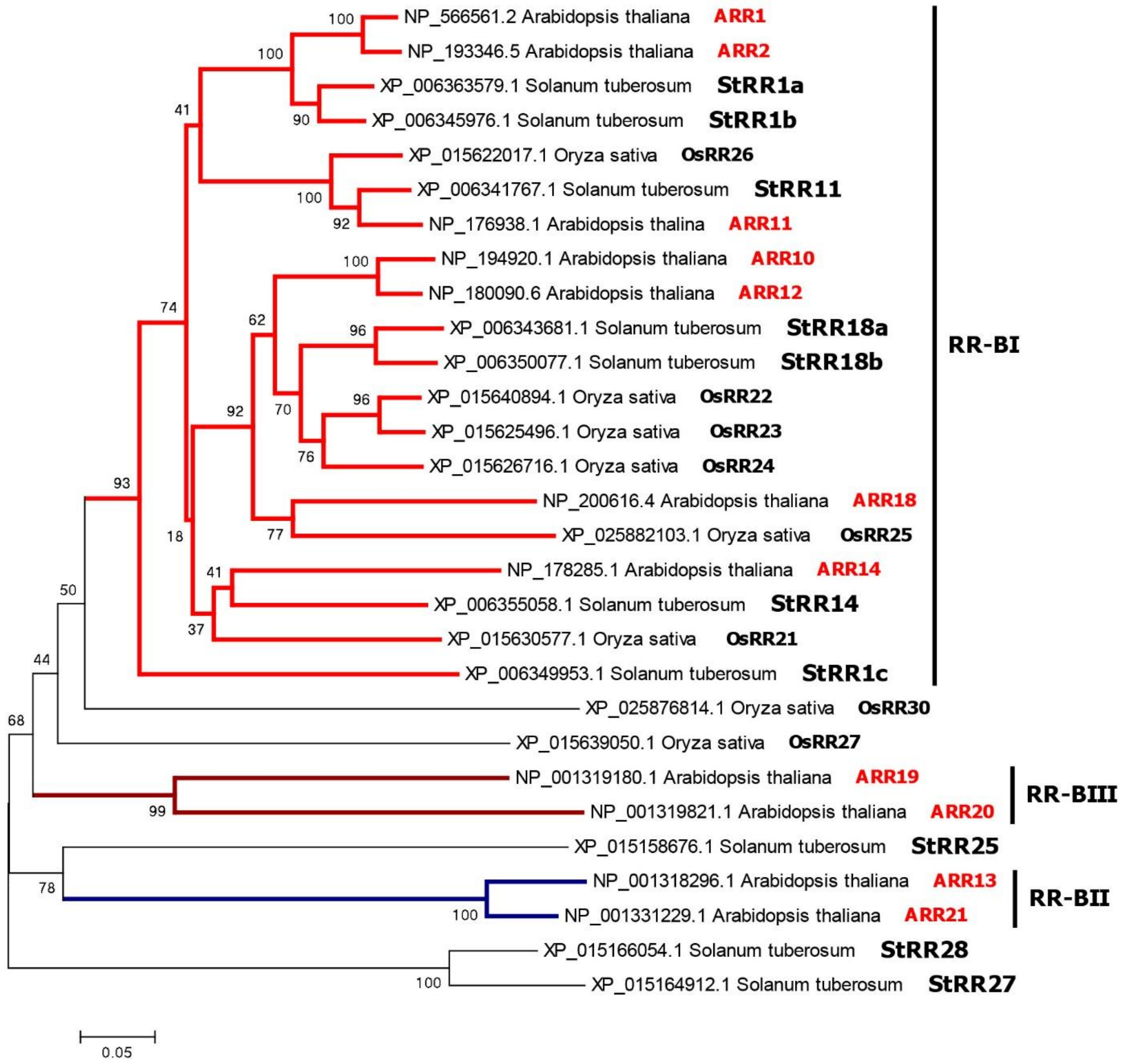

Fig. S2A. Evolutionary relationships of RR-Myb taxa.

The evolutionary history was inferred using the Neighbor-Joining method [1]. The optimal tree with the sum of branch length = 4,61453618 is shown. The percentage of replicate trees in which the associated taxa clustered together in the bootstrap test (100 replicates) are shown next to the branches [2]. The tree is drawn to scale, with branch lengths in the same units as those of the evolutionary distances used to infer the phylogenetic tree. The evolutionary distances were computed using the p-distance method [3] and are in the units of the number of amino acid differences per site. The analysis involved 29 amino acid sequences. All positions containing gaps and missing data were eliminated. There were a total of 107 positions in the final dataset. Evolutionary analyses were conducted in MEGA6 [4]. For the construction, only the gene receiver domains were used due to the complexity of the structure of proteins. Pseudo RRs were excluded, since in their presence the tree structure becomes statistically unreliable.

1. Saitou N. and Nei M. (**1987**). The neighbor-joining method: A new method for reconstructing phylogenetic trees. *Molecular Biology and Evolution* **4**:406-425.

2. Felsenstein J. (**1985**). Confidence limits on phylogenies: An approach using the bootstrap. *Evolution* **39**:783-791.

3. Nei M. and Kumar S. (**2000**). *Molecular Evolution and Phylogenetics*. Oxford University Press, New York.

4. Tamura K., Stecher G., Peterson D., Filipski A., and Kumar S. (**2013**). MEGA6: Molecular Evolutionary Genetics Analysis version 6.0. *Molecular Biology and Evolution*30: 2725-2729.

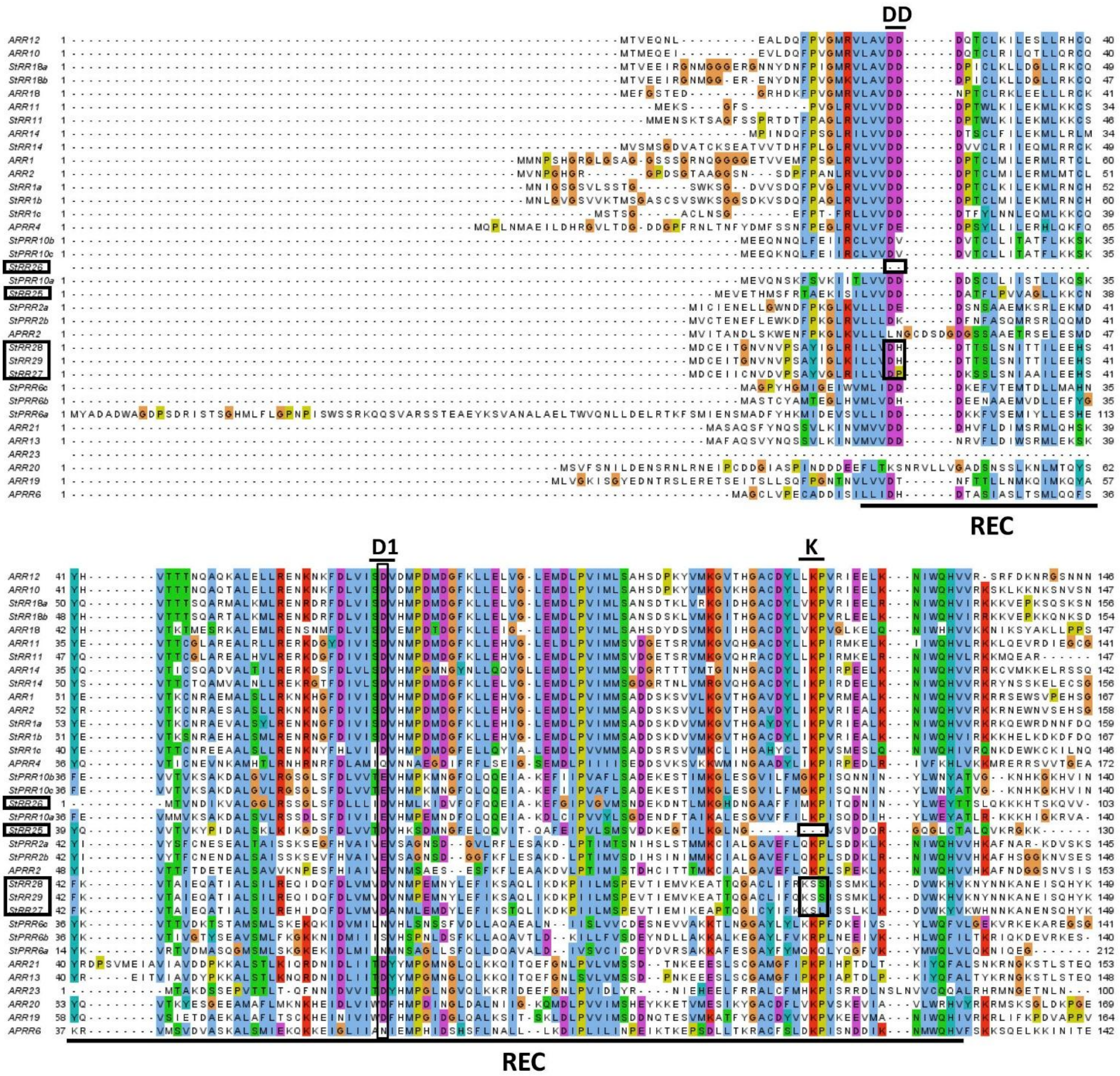

Fig. S2B. Alignment of RR-Myb proteins.

Noticeable abnormalities in structure of potato specific RR-Myb`s are framed.

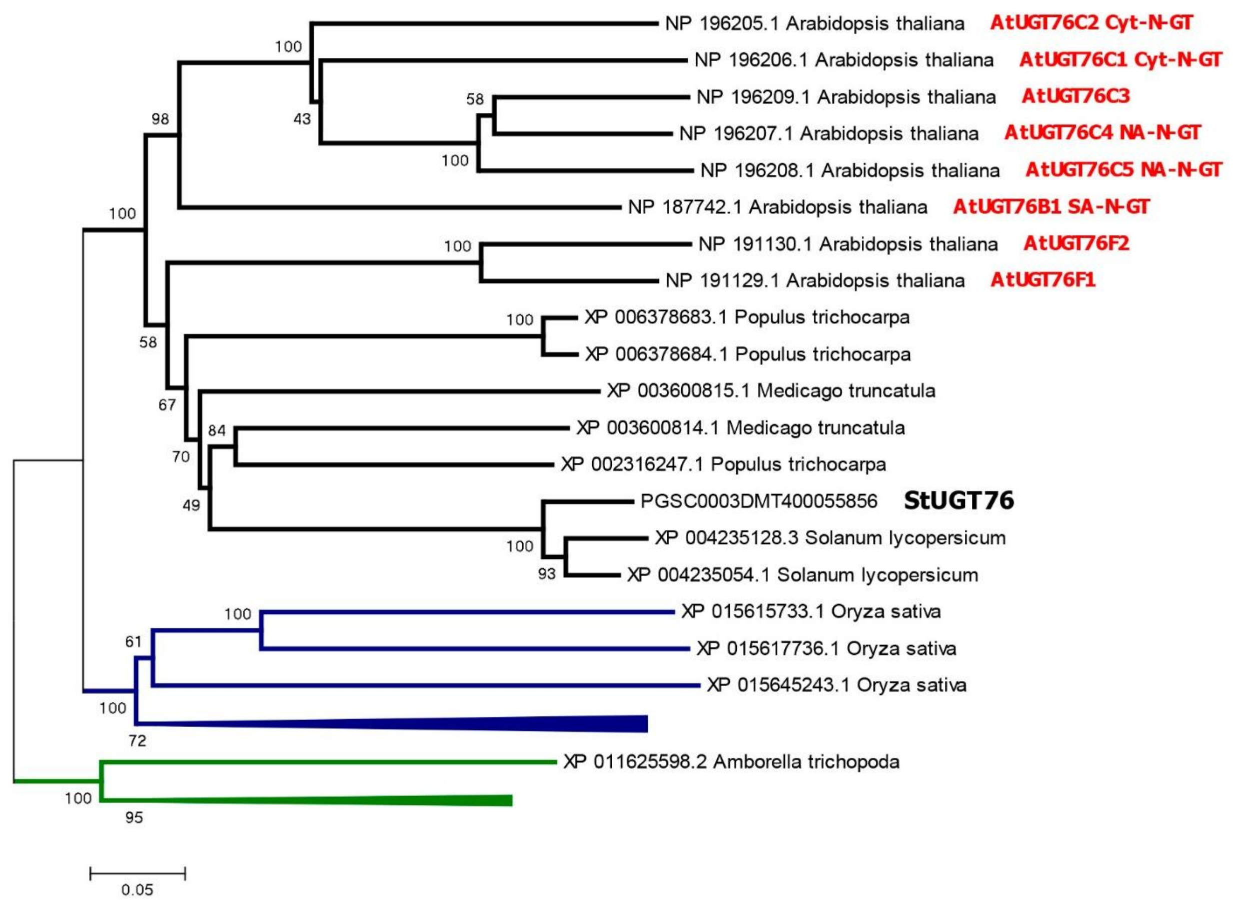

Fig. S3A. Evolutionary relationships of taxa UGT73.

The evolutionary history was inferred using the Neighbor-Joining method [1]. The optimal tree with the sum of branch length = 5.46637192 is shown. The percentage of replicate trees in which the associated taxa clustered together in the bootstrap test (100 replicates) are shown next to the branches [2]. The tree is drawn to scale, with branch lengths in the same units as those of the evolutionary distances used to infer the phylogenetic tree. The evolutionary distances were computed using the p-distance method [3] and are in the units of the number of amino acid differences per site. The analysis involved 28 amino acid sequences. All ambiguous positions were removed for each sequence pair. There were a total of 584 positions in the final dataset. Evolutionary analyses were conducted in MEGA6 [4]. The tree is built according to full-length protein sequences, since a single domain occupies almost the entire protein. For references, see legend to Fig. S2A.

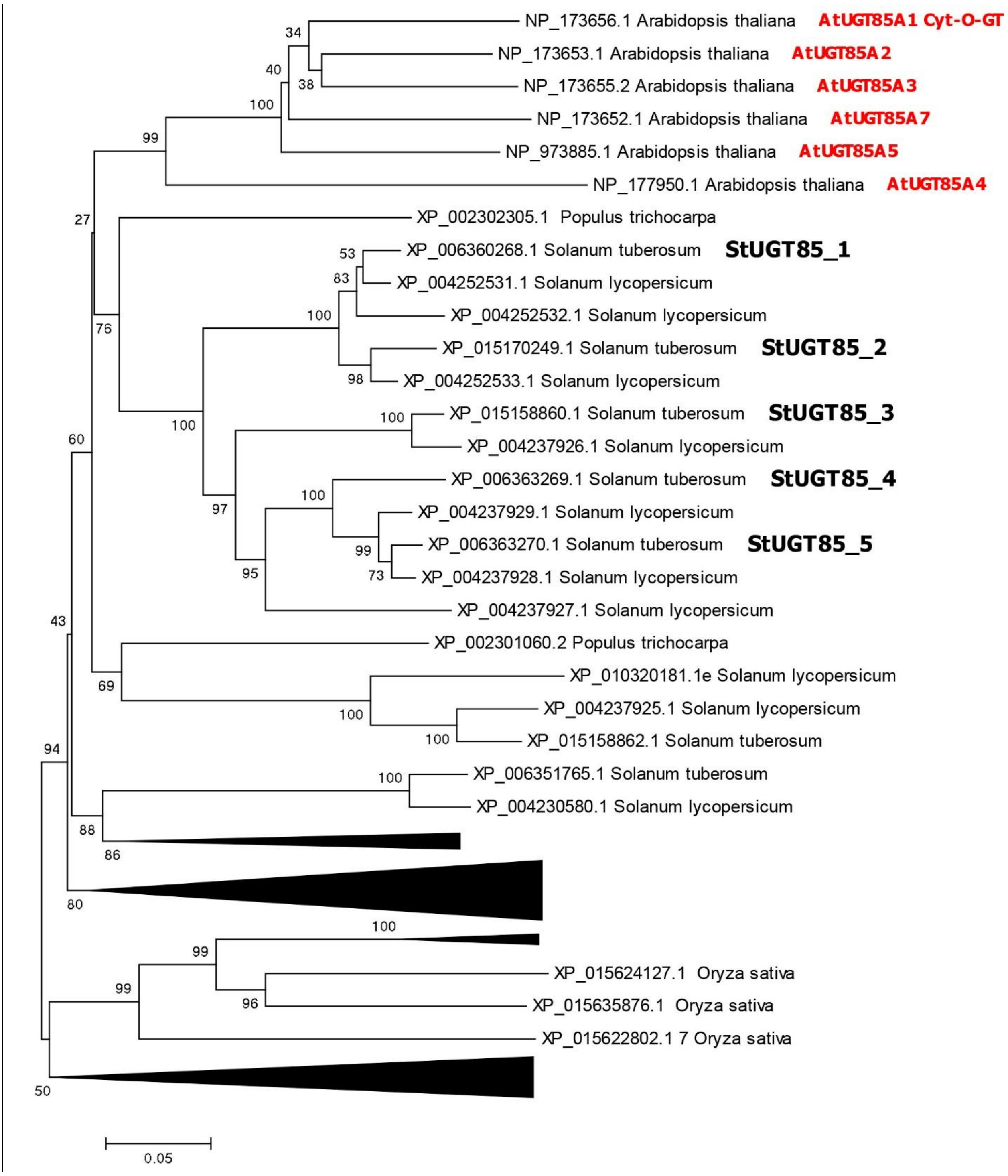

Fig. S3B. Evolutionary relationships of taxa UGT85.

The evolutionary history was inferred using the Neighbor-Joining method [1]. The optimal tree with the sum of branch length = 7.09956765 is shown. The percentage of replicate trees in which the associated taxa clustered together in the bootstrap test (1000 replicates) are shown next to the branches [2]. The tree is drawn to scale, with branch lengths in the same units as those of the evolutionary distances used to infer the phylogenetic tree. The evolutionary distances were computed using the p-distance method [3] and are in the units of the number of amino acid differences per site. The analysis involved 75 amino acid sequences. All positions containing gaps and missing data were eliminated. There were a total of 391 positions in the final dataset. Evolutionary analyses were conducted in MEGA6 [4]. The tree is built according to full-length protein sequences, since a single domain occupies almost the entire protein. For references, see legend to Fig. S2A.

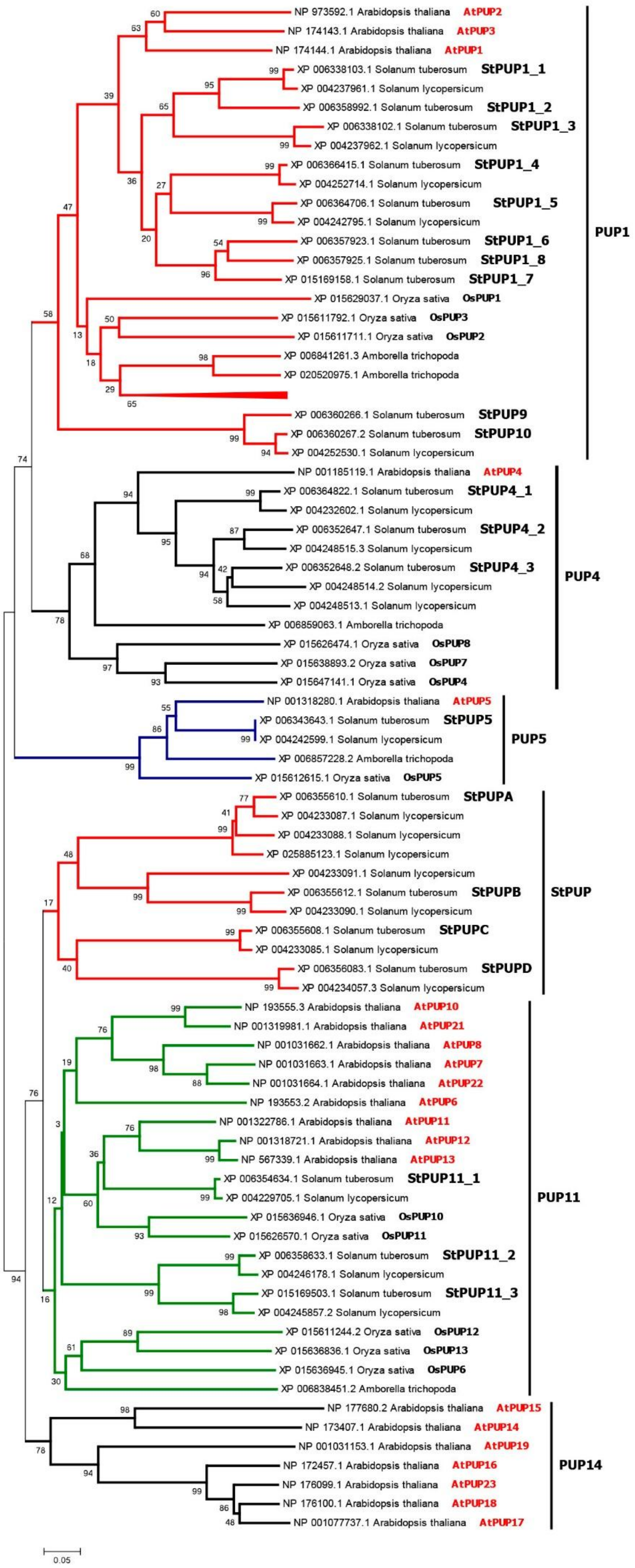

Fig. S4. Evolutionary relationships of taxa PUP.

The evolutionary history was inferred using the Neighbor-Joining method [1]. The optimal tree with the sum of branch length = 13.22737636 is shown. The percentage of replicate trees in which the

associated taxa clustered together in the bootstrap test (100 replicates) are shown next to the branches [2]. The tree is drawn to scale, with branch lengths in the same units as those of the evolutionary distances used to infer the phylogenetic tree. The evolutionary distances were computed using the p-distance method [3] and are in the units of the number of amino acid differences per site. The analysis involved 83 amino acid sequences. All positions containing gaps and missing data were eliminated. There were a total of 66 positions in the final dataset. Evolutionary analyses were conducted in MEGA6 [4]. The tree is built according to full-length protein sequences, since a single domain occupies almost the entire protein. For references, see legend to Fig. S2A.

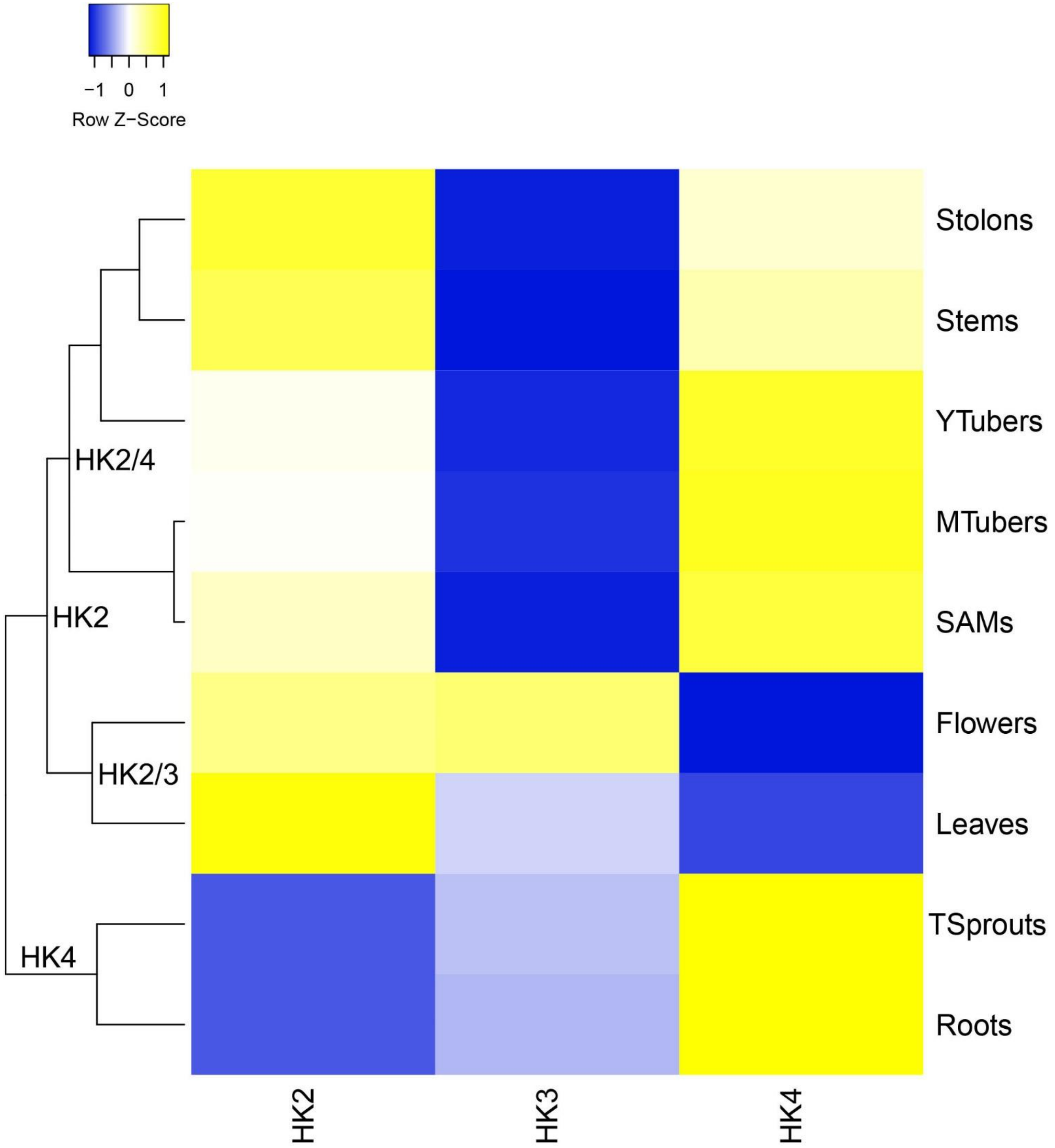

Fig. S5A. Cluster analysis of StHK expression in different potato organs performed by Heatmapper (http://www.heatmapper.ca/expression/).
Scale Type – Row, Clustering Method – Average Linkage, Distance Measurement Method – Euclidean. Organ/tissue designations are the same as in Fig. 1.

Babicki S., Arndt D., Marcu A., Liang Y., Grant J.R., Maciejewski A., Wishart D.S. Heatmapper: web-enabled heat mapping for all. Nucleic Acids Res. 2016, 44 (W1): W147-W153. doi:10.1093/nar/gkw419

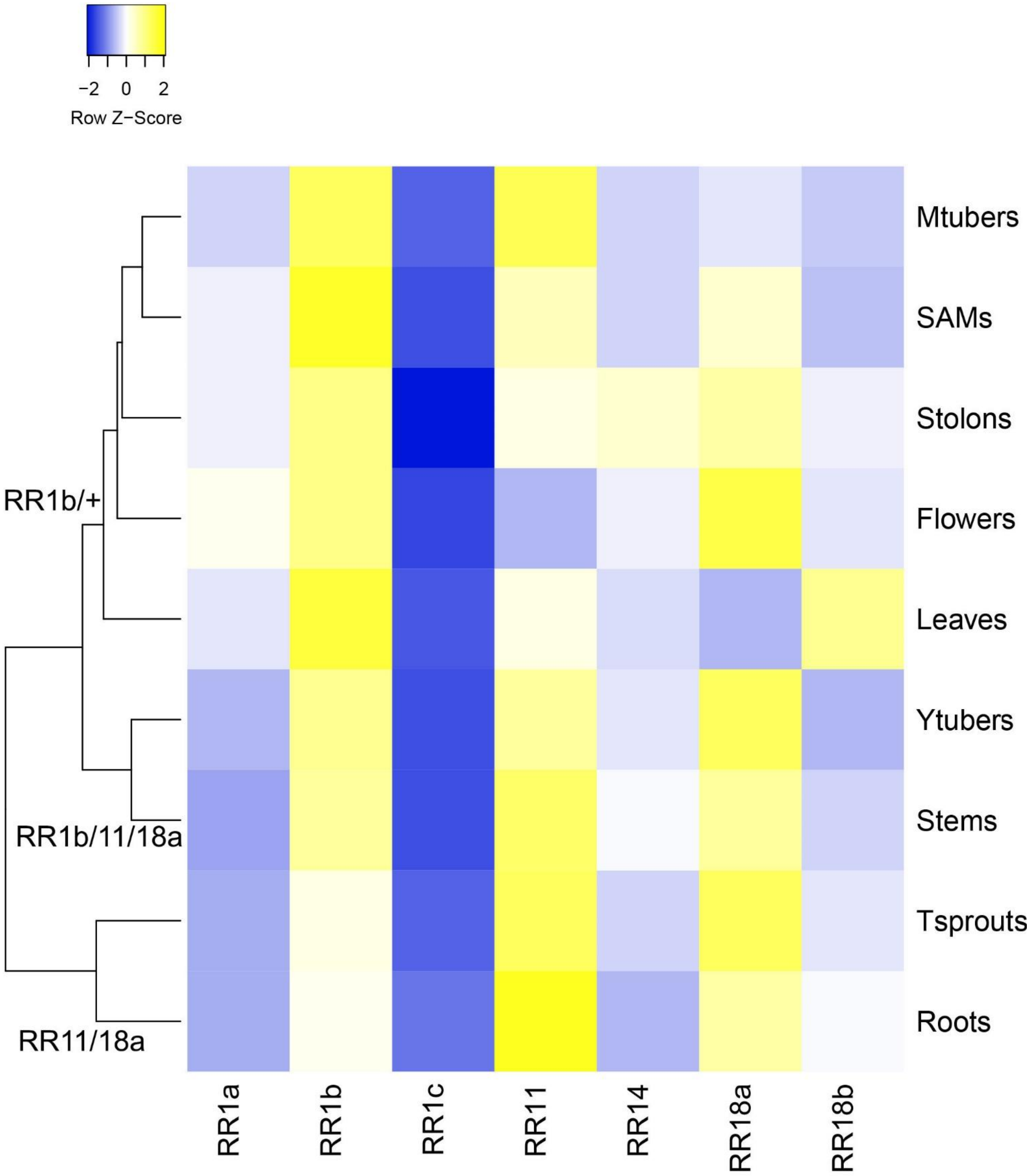

Fig. S5B. Cluster analysis of StRR-B expression in different potato organs performed by Heatmapper (http://www.heatmapper.ca/expression/).
Scale Type – Row, Clustering Method – Average Linkage, Distance Measurement Method – Euclidean. Organ/tissue designations are the same as in Fig. 1
.
